# Hospitals and the State

**Published:** 1912-09-28

**Authors:** 


### Hospitals and the State.

### SIR WILLIAM COLLINS' PAPER AND THE DISCUSSION.

The outstanding characteristic of the present age is the tendency to discuss fundamentals. The spirit of unrest, alike in international and national relations, is abroad. Old dynasties and Oriental systems, dating from hoary antiquity, are doomed and decadent or have passed away before our eyes in bloodless revolutions. Home politics at every turn are engrossed with elemental questions which have been undisturbed for generations. The seat of sovereignty, the ethics of rebellion, the foundation of law and order, the basis of the franchise, majority rule—its rights and duties jostle one another in almost daily disputations. The limits, if any, of State intervention, the inalienable rights, if any, of the individual personality, as our forefathers were in the habit of defining them, have been obscured or obliterated. Herbert Spencer's doctrines formulated in his "Man *versus* the State," or John Stuart Mill's views "On Liberty," deemed in their day the final word in political philosophy, are accounted back numbers. Under pressure from apostles of Socialism and Syndicalism, or in obedience to the gospel of Eugenics, State intervention, inspection, and regulation are welcomed into, rather than warned off from, almost every region of human activity. In matters of religion and conscience alone is any tendency in an opposite direction to be detected, and here, as an exception to the rule, do we encounter a tendency to renounce State establishment or State endowment and a resistance to public subsidy or to the requirement of denominational teaching.

#### "Voluntaryism or Communism?"

Your Association, I understand, exists to consider how best the care of the sick and injured should be managed and administered in a well-ordered community. Many and attractive are the details and subsidiary subjects which must invite and claim your earnest consideration; but at the present time, more than at any other within living memory, the fundamental question of Voluntaryism or Communism in the matter of hospitals is challenging public attention and engrossing the consideration of all thoughtful reformers.

We have arrived at this stage, half unconsciously, as the result of a series of legislative enactments, nearly all making in one direction, but as is our wont in this country without any very clear preconception of the end in view, and often accompanied by ingenuous disavowals of any socialistic trend on the part of the authors of the legislation in question.

The last century has witnessed the transfer from private ownership and management to State or municipal ownership of many services—often with great advantages alike to the services and to the community served.

The stories of our prison service, street lighting, fire prevention, water supply, gas and electric supply, cemetery provision, and tramway service would each afford evidence of transfer from private to public hands.

The history of the Poor Law both prior to and since 1834, as told in the voluminous Reports of the recent Royal Commission, supplies additional examples of the same drift. Sickness—the Nemesis of neglect and the bed-fellow of poverty—has ever made Lazarus an occasion of concern, if not an object of compassion, to the self-complacent Dives. Poverty and disease—those twin stars of evil omen—revolve the one around the other in vicious circulation. Thus sickness *plus* destitution, no longer cared for as in earlier days by the Church, became a public charge and has been long quartered upon the rates. Infectious disease, by reason of its peril to others, was deemed a fit subject for public intervention, and even when unaccompanied by destitution its care was accepted as properly a matter for public control and expenditure. The principle was extended to medical cases, other than infectious, and surgical cases. As the Report of the Royal Commission on the Poor Laws correctly puts it (par. 87, p. 256): "The voluntary hospitals are doing a great work, which, if they did not exist, would have to be performed by the Poor-Law authorities, or by some other public authorities charged with the provision of hospital accommodation;" and in a later paragraph (111, p. 262) it is added: "The sanitary authority may either build a hospital or contract for the use of one or combine with another sanitary authority in providing a common hospital. There is no limitation either to the diseases which may be treated in such hospitals or to the classes of persons who may participate in their benefits. Accordingly a town council may at any time erect a general hospital for the use of the inhabitants of the borough, no matter from what disease they may be suffering." The voluntary hospitals at the present time, therefore, supply a want which in their default would fall upon the rates. Such incidence is rare, but it is well to remember that the fact of the continuance of voluntary hospitals and their beneficent work is not owing to the absence of legislative provision for the services they render being supplied out of rates.

So also with disease of the mind; when its victims were suffered to shock the public gaze they were thrust into some Bridewell or Bedlam lest they should prove a "public scandal to the high displeasure of God and damage to the King's people." Out of that non-altruistic motive has grown, under the teaching of Pinel, of Tuke, and of Connolly, that solicitous regard for the insane for which such palatial provision is now made out of public funds all over the country. Similarly, reformatories and inebriate homes have, under public authority, been made available for those whose vicious indulgence has brought them under the cognisance of the Courts; and Parliament is busy with further arrangements conceived for the benefit of the "feeble-minded."

### The Legislature's Assault on the Hospitals.

This "stream of tendency," which we in our time have witnessed in legislation and administration, has flowed around, and as it were engulfed the so-called voluntary hospitals, and has forced on many minds some heart-searchings as to the future of those great institutions, some of ancient foundations, and all, with rare exceptions, performing immensely valuable service to the public whose confidence they have justly earned, and whose voluntary support they have richly deserved. Moreover, several of the most distinguished of our hospitals in our large towns have, since the old apprentice system fell away, become intimately connected with the education of the medical profession; for while a hospital can very well exist without a school, a medical school, nowadays, can hardly exist without a hospital.

From yet two other sides has the Legislature led up to what almost amounts to an assault upon the voluntary hospital as they have been known for the last two or three generations. In reciting some of the services, formerly voluntary and vested in private hands, but now publicly maintained by State or rate, I did not include Education. It was a reservation rather than an omission. No service has in the last half-century received so much public attention, and probably in no other case has so great and so rapid a transition from Voluntaryism to State care been effected. Witness the paradox of Wilhelm von Humboldt, who taught that "Education lies quite outside the limits within which Government should confine its operations," and then proceeded to falsify his doctrine as Director of Public Education in Prussia. As I said in my Presidential Address at the International School Hygiene Congress in 1907: "You may regret that the Education Act of 1870 was the first step in what may be denounced as a socialistic departure. The State has either gone too far or it has got to go further; and, if the latter, then, in the case of dire necessity, it will have to put its stately hand into the State pocket and administer, grudgingly or otherwise, State aid. How to do these things without over-riding parental rights or undermining parental responsibility is a problem of the greatest delicacy and difficulty. Here, as in many other instances, humanity and liberty, the State and the individual, appear to assert rival claims, and their due adjustment will continue to tax the power and the skill of the twentieth-century statesmen."

### Education, Meals, Inspection.

The State by the Acts of 1870 and 1876 in compelling children between the ages of five and fourteen to attend school for five hours daily for 200 days in the year has made itself to some degree a co-partner with the parents in the welfare of children attending public elementary schools. The result has been to bring child life for those nine years under public review. Reformers and zealots of all sorts have been anxious to seize upon this organised school life as affording opportunity for all kinds of physical, mental, moral, and spiritual exploitation. The democratic House of Commons, elected in 1906, passed the Provision of Meals Act, and in drafting the Report of the Committee on that Bill I drew attention to these very considerations to which I have been referring. Hot on the heels of that Act came the Education (Administrative Provisions) Act, 1907, under which local education authorities were required to provide for medical inspection, and were empowered to make arrangements for " attending to the health " of the children attending school. Both of these new services were introduced and pressed forward as being auxiliary to educational fitness, and as arising naturally out of the reponsibilities already undertaken by the State in compelling attendance at school.

In the House of Commons and before the Medico-Legal Society I have directed attention to this recent legislation because it serves to show in the first place how easily, and almost unwittingly, fresh services are shouldered by the State or amiably imposed by it upon local authorities, and in the second place they exhibit in a striking and significant manner the tendency to supersede voluntary organisations while paying homage to them and nominally invoking their co-operation. The shifting of individual and social duties on to the shoulders of a State or a municipal department is as facile as is the subsequent expostulation at bureaucracy and officialdom to which such abnegation of duty and responsibility inevitably leads.

Thus the Education (Provision of Meals) Act, 1906, directed that there should be associated with the local authorities certain voluntary committees through which provision should be made for feeding children attending school who were by reason of insufficient nourishment unable to take full advantage of their education. These " Canteen Committees," as the Act calls them, have developed into " Care Committees," not for feeding only, but for all and sundry purposes. The voluntary organisations have largely gone to the wall; food as well as the appliances has in many cases been paid for out of rates; and there is a strong movement in favour of feeding the children through this agency during holidays as well as in term time. It thus is coming about, as I have on several occasions ventured publicly to foreshadow, that the age of five to fourteen is being regarded not so much a period for " instruction " as a period for the State regulation and maintenance of childhood. This may by reason of parental neglect be, and doubtless is in many instances, greatly to the benefit of the child's well-being, but it must, nevertheless, tend to weaken and nullify those appeals to parental responsibility and the fostering of good home influences, on which we are always bidden to fix our attention as the true objective in the ideal State.

A brief study of what is going on in regard to medical inspection and treatment of school children under the vague and casual powers of the Act of 1907 brings us up against the whole problem of the future of voluntary hospitals, and that is why I would specially invite your consideration of it.

### A Parliamentary Retrospect.

I well remember the first debate in the Commons on the subject in 1906 initiated by Mr. Tennant, M.P., when that gentleman stated that he was " particularly careful to guard against medical treatment—periodical medical inspection he held did not include medical treatment." Moreover, Mr. Birrell, then President of the Board of Education, said: " It was not in his mind that there should be medical treatment." Dr. Macnamara said: " Not treatment—inspection." The President replied: " But continuous inspection would mean treatment," to which Dr. Macnamara retorted: " If there is continuous medical inspection there are many agencies which might be brought into play to take over the treatment." Accordingly, in the following year, when these proposals came to be enacted a proviso was added in these words: " That in the exercise of powers under this section the local education authority may encourage and assist the establishment or continuance of voluntary agencies and associate with itself representatives of voluntary associations for the purpose." The Board of Education in a Circular (596) laid it down that " before direct treatment of ailments is undertaken by a local education authority itself by means of a school clinic or otherwise, all reasonable advantage should be taken of the benefits of such institutions as hospitals and dispensaries," and that " only those children shall be treated in a school clinic for whose treatment adequate provision cannot otherwise be made, whether by the parents, or by voluntary associations or institutions such as hospitals, or through the agency of the Poor Law," and thus the Board sought to " stimulate a healthy economy in the use of resources, particularly by that obtained by wise co-operation between local education authorities and voluntary agencies. . . ." And in doing so was " guided by the apparent intention of Parliament." In short, " other available means of providing treatment should have been exhausted as far as possible " before resorting to school clinics established and maintained out of public funds.

### Three Reports and the Moral.

The Chief Medical Officer of the Board, from whose first Report (for 1908) these extracts are taken, in his account of the work done by local authorities in respect of medical treatment very properly emphasised the fact that they were acting under a statute which gave them a power " which they may or may not exercise," whereas in the case of medical inspection a statutory " duty " was laid upon all education authorities.

The second Report of the Chief Medical Officer of the Board indicated a tendency to water down the advice originally vouchsafed to resort to hospitals and such like agencies before establishing school clinics out of rates, and assumed a rather apologetic tone in justification of the original advice given by circular to education authorities to avail themselves of such voluntary institutions. He points out that "voluntary hospitals have been in receipt of State aid (in the way of subscriptions) both from Poor-Law unions and municipalities for many years past, in order that the poor might obtain adequate treatment without payment." And again: "Although in adopting this course (resort to hospitals rather than establishing school clinics) the Board have laid themselves open to some criticism, the course has, in fact, continued to receive increasing support, as one commending itself to sober public opinion. After all, these voluntary hospitals were established, and are at present maintained for the medical relief of the poor. That the State has called upon the local authorities to organise machinery for detecting the ailments of school children, many of whom are poor and necessitous, does not therefore make the claim of these children upon these institutions any the less in principle, though it rapidly becomes wholly different in degree . . . but it does not mean that voluntary hospitals should not still be the refuge of poor children who need medical treatment. . . . The Board were not in a position under the Act, as many seem to have supposed, to *require* local education authorities to carry out the treatment of children found to be defective. . . ."

In the third Report of the Chief Medical Officer of the Board there is a remarkable further and more striking shifting of position. Treatment and inspection are rolled together, and the hospital is pushed into the background while the school clinic gains in favour. Thus we read: "In fine, a school medical treatment scheme to have permanent value must not be an *ad hoc* arrangement tacked on, as it were, to the organisation for medical inspection, but the arrangements for the inspection and treatment of the child respectively must form one co-ordinated service." And as for hospitals the later view, which amounts almost to a *volte face*, is that their "place should be in the main a subsidiary one." "If used at all, both space and service must be set aside for the exclusive use of the children from school," and "in short, the method of the school clinic must be reproduced wherever and however treatment of school children is conducted." These changes of front on behalf of the central department, so bewildering alike to hospital boards and to local authorities, may be the result of outside pressure or criticism, but are hardly the outcome of mature consideration, and are perhaps the natural sequel to rather hasty and slovenly legislation. Speaking in the House of Commons on April 23, 1909 (before any of the three Reports I have quoted had seen the light), I said: "What are we going to do with the results which medical inspection has exposed to view? When the question of continuous medical inspection was discussed in this House [July 16, 1906] with the prospect of medical treatment provided out of public funds, I remember the President of the Board of Education (Mr. Birrell) entirely dismissed the notion that medical inspection was going to lead to medical treatment . . . but those who are working on educational authorities know very well that very delicate questions are arising as to what is to be done with these cases of physical defect and disease, which are exposed as the result of medical inspection—whether they are to be referred to hospitals; whether the parent is to be required to have the necessary treatment carried out; or whether school clinics and municipal hospitals ought to deal with them. That is a question calling for most serious investigation at the present moment, and before any legislation takes place I hope some committee or commission will be set up to look into the whole question thoroughly. It is an important question, and it was rather airily dealt with by the House of Commons when it passed the clause which empowered the local authority 'to attend to the health of the children'—a very vague phrase, and I think further consideration has shown how indefinite it is, and how uncertain it is, what it really covers. The Act is very particular as to the dates at which medical inspection should take place, but it is exceedingly indefinite as to what is meant by 'attending to the health of the children.' This question requires very much more careful consideration and treatment than it has yet received, and may require not only administrative, but legislative, change." On December 2 of the same year I again, by question, pressed Mr. Runciman, then President of the Board, to set up such an inquiry, but he replied: "I think it is desirable to postpone a reply to my hon. friend's question pending the issue of the first Report of the Medical Department of the Board which is now in an advanced state of preparation." Since then we have witnessed the issue of the second and third, as well as the first, of these Reports, and they serve not only to accentuate the need for such investigation but also exhibit the vacillations of the department which have been the result of its neglect. Thus we go muddling along; acting first and inquiring afterwards, or allowing sloppy legislation to be interpreted variously by different officials at different times or at different times by the same officials.

By an Act passed in 1909 power was given to local authorities to recover from parents the cost of medical treatment for which the authority had arranged. I doubted the wisdom of this proposal, but secured the introduction into this Act of a proviso to the effect that a parent was not compelled to submit his child to such treatment or inspection. This seemed desirable since some authorities have unwisely threatened parents under the Children Act, 1908, with proceedings for "neglect" if they failed to provide such "medical aid" as the education authority prescribed. It would be anomalous indeed if the parent of a child of school age treated at a hospital, by arrangement with a local authority, for some defect revealed by inspection were proceeded against for recovery of cost of treatment when simultaneously that hospital opened its doors for free treatment of children under five or over fourteen, or, indeed, for children of school age who might present themselves otherwise than through some arrangement or contract made between the hospital and the authority. This attempt to charge for hospital treatment introduces an element of difficulty, but there is no reason why the education authority, like the Poor-Law authority, should not subsidise voluntary hospitals, not by capitation fees, but in block grants for work done which, in default of hospitals, would fall on the authorities in question. As the second Report of the Chief Medical Officer of the Board correctly states: "It is incorrect to suppose that subsidy from the education authority to a hospital is a new departure or creates a new precedent of State support to general hospitals." Mr. Pease, the new, and fourth, Education Minister of the present Government, on June 6 announced a grant of £60,000 with an intimation that there was more to come in future years "to help and encourage education authorities in treating these ailments" of suspected school children.

### The Insurance Act.

The other direction from which recent legislative action will lead up to a review, if not a revision of, our voluntary hospital system, is of course the National Insurance Act. It is not easy to forecast the influence of this Act upon the hospitals, but it scarcely requires the spirit of prophecy to foretell that it cannot be without influence. An attempt was made last year by the King's Hospital Fund, based on such materials as were available, to forecast the probable results. From this it appeared that the number of in-patients would not improbably be increased, by reason of the more systematic medical observation of the industrial population, even though the number of tuberculous cases admitted to hospital might eventually be reduced. Out-patients, on the other hand, might tend to be of a different class to those hitherto dealt with, and might not improbably be reduced in numbers. There appeared to be widespread apprehensions that contributions to hospitals, alike from employers and employed, would be reduced. The power given under section 21 of the Act to approved societies or Insurance Committees to subscribe to hospitals was not thought likely to operate very beneficially. If such contribution is optional it is not likely to be large, and if compulsory it was apprehended that this must lead to intervention in management.

It is clear that a not inconsiderable number of members of Friendly Societies take advantage of hospital treatment; any male hospital ward will probably be found to contain several such members or insured persons. The suggestion that the sick benefits of such should be paid as *per capita* contributions to the hospitals admitting them, though it sounds simple and attractive, will on further examination be found to present numerous difficulties.

As I ventured to point out when the Bill was introduced, and when on all sides there were expressions of approval of its amiable principles, that almost every detail raised a principle, and that a rather radical imperfection was embodied in the claim that the Bill would provide all insured persons with full medical and surgical treatment, whereas the only means for supplying such benefits, in other than tuberculous cases, was to be by way of arrangement of Insurance Committees with individual general practitioners. The fact that the services of the hospitals were largely resorted to by general practitioners for persons of the insured or insurable class was either ignored, or it was tacitly assumed that they would be relied on in the future to discharge such benefits to the community, possibly even in a greater degree than before. The recent announcement that the anti-tuberculosis crusade is to be a national one, and not confined to insured persons under the Act or their dependents, will doubtless raise, in a similar way to the treatment of school children and of insured persons, the *rôle*, if any, which the voluntary hospitals are to play. If the arrangements set up by public funds are outside the work of the hospitals an artificial severance of tubercular disease in its protean manifestations will result in much duplication of machinery both clinical and pathological. As we are assured by some authorities that 70 and more per cent. of the population of our large towns are at some time or other tuberculous the outlook is not free from perplexity!

In Germany, where free treatment in hospitals of the compulsorily insured has a recognised place, it is interesting to observe what results have followed, especially in regard to clinical teaching.

Dr. Friedrich von Müller, Professor of Clinical Medicine in Munich, informed the Royal Commission on University Education in London on June 30, 1911, that "in Germany every workman and workwoman and all domestic servants are compulsorily insured against disease. In case of illness or accident they have the right to free treatment and admission to a hospital, and the Insurance Fund is obliged to pay to the hospital for every patient admitted a certain sum, averaging 3s. a day. . . The hospital patients are therefore not admitted by charity, and our hospital work is therefore not so much charity work as in England. Most of our patients have a *right* to be admitted, and they cannot be discharged till they are cured, or till their time is over. This is the reason why our patients are not willing to be examined by the students, or to be made the subjects of demonstration in the theatre."

### Its Effect on Medical Education.

Professor Müller explained that under this system four-fifths of the support of some hospitals is now derived from the Insurance Fund. The effect on medical education is considerable and remarkable. In Berlin the Municipality does not allow the professors to enter the hospitals. Professor Müller stated that in Munich he has to ask permission of each patient before using the case for instruction. "Not only can they refuse, but they do." Alluding to the insured patient the Professor naïvely says: "He is perfectly free; he is not compelled at all to become clinical material. . . . It is his right to be taken in because it is a public institution, and it is not at all his duty to be used for education." The Professor was constrained, however, to admit that "the practical instruction in England is better."

If we are to follow methods made in Germany, and if a contribution *per capita* is to be made to hospitals in respect of insured persons treated therein, in view of the fact that a portion, at any rate, of such contribution comes from the pocket of the insured, similar claims will doubtless be advanced here and medical education may be accordingly affected. The compulsory contribution will reciprocally give rise to assertion of rights and the refusal to be used as "clinical material." Under a voluntary system such assertion is absent, not because patients are regarded with less personal consideration here than in Germany—the reverse is notoriously the case—but because the spirit of compulsion profoundly alters the whole relationship between the medical man and his patient.

As regards the hospital staff, I suppose it is generally agreed that under a State or municipal service the visiting medical officers could not, or would not, any longer perform their duties in an honorary capacity. Whether the same class or type of visiting physicians and surgeons would be secured under a salaried system, controlled by a publicly appointed committee, is to say the least problematical. It has been estimated that if the hundred odd voluntary hospitals of London were put on the rates and staffed and managed after the fashion, say, of the Metropolitan Asylums Board institutions, it would mean a new charge of something like 8d. in the £ on the rates. On the other hand, the non-medical staff and those medical officers in receipt of salaries commensurate with service might fare better in the matter both of pay and pension. As one who succeeded in piloting through Parliament an Act to secure contributory pensions for asylum officers, I know how strong is the desire for somewhat similar treatment of the case of hospital officers. Under a State system it would no doubt be far easier to establish and maintain some such universal system of superannuation.

A valuable Report has just been issued by the King's Hospital Fund, prepared by Lord Mersey and the Archbishop of York, on "the system prevailing in the London hospitals with regard to the admission of out-patients." The Commissioners state that they have not considered the possible effect of the National Insurance Act on the out-patient question; they, however, agree that "the use of the out-patient departments by the well-to-do is not common," though their use by "persons able to pay small fees to doctors probably exists to quite an appreciable extent." They look to the development of the almoner system, and to the utilisation of out-patient departments for consulting rather than for therapeutic purposes, as valuable safeguards against such abuse.

Difficulty, however, will always be met with in dealing with (1) the urgent cases, which must be admitted irrespective of poverty, and (2) interesting clinical cases or cases of doubtful diagnosis which will be franked, or will demand elaborate equipment which the general practitioner cannot be expected to supply. We hear of royal personages, very wisely, going into hospital rather than being treated even in a palace, and if accompanied by voluntary contributions it is difficult to refuse to permit the exercise of a similar preference in humbler folk. In short, classification of patients on a basis of ability to pay a practitioner has no logical relationship to classification according to urgency, difficulty of diagnosis, a need for specially skilled or expert treatment—that is to say, according to suitability for hospital or non-hospital treatment.

### Where the State Should Walk Warily.

This question of the abuse of out-patient departments, however, sinks into relative insignificance beside that of Voluntaryism or a State system for treatment of the sick and injured. Recent legislation has, as I have shown, brought that question into the foreground. Is it to be answered by the application of principles or by mere eclecticism or empiricism? Is the presumption to be in favour of the State or of these charities remaining in private hands? The care and control of infectious disease, and of lunacy requiring restraint, are clearly distinct from other cases of illness and cases of accident; State intervention in such cases may be justified on principles of which individualists would not disapprove. But, in other cases, a State system is beset with many difficulties. Many evidences suggest that the State should walk warily in dealing with matters of health—physical, mental, or moral. The appearance of the Peculiar People in our Law Courts, and their conviction and punishment for relying on Scripture rather than on modern medical science does not afford an edifying spectacle. The cult of Christian Science, by some regarded as a revolt against a too pontifical bearing of medical orthodoxy, is a significant development of recent origin. The surrender in turn of each of the great political parties in the State to the claims of the conscientious objector in the case of vaccination furnishes food for thought. These facts indicate that in matters of health, as in matters of religious education, there is a region from which the power of the State is warned off. The "free choice of a doctor," of which much has been heard in connection with the Insurance Act, is corollary to this notion. The experience in Germany of the refusal of the compulsorily insured, when in hospital, to be regarded as "clinical material" is also significant. We have indeed of late seen how "the individual withers and the State is more and more." What the State can, and should, undertake to do has been variously decided by thoughtful men from Plato to Lloyd George. The flood-tide of State Socialism has before now ebbed and Individualism has violently or peaceably reasserted itself. As with education, which Humboldt declared to be wholly outside the ambit of State action, so with health, Spencer, Mill, and F. W. Newman could be quoted to a like effect. At the present time both are favourite subjects for legislative experiment. Such experimentation may be overdone, and in a fit of revulsion even good State or municipal work may be set back or sacrificed.

On the other hand, I would put in a plea to the effect that good voluntary work such as that which has been successfully organised in this country in the case of our general and special hospitals, which have supplied a felt want, have won the confidence of the public, inspired disinterested professional service among the most capable of the profession, and kept the healing of the sick—a calling almost divine—from being impersonalised and stereotyped into routine and officialdom, should not be lightly thrown away through a mere careless acquiescence in a communistic trend.

### The Discussion.

The discussion was opened by Dr. George Auden, school medical officer, Birmingham.

It is no purpose of mine, he said, in these hurried notes to extenuate or defend the position to which successive legislation has brought us; but I shall attempt to describe the efforts which are being made in Birmingham to deal with the problem of ill-health amongst the children in attendance at the elementary schools, and to show how these are being or may be linked up with the voluntary agencies which are working in the same direction.

Few persons will be prepared to deny that the object of education is the production of the efficient citizen. Now, for this purpose, the mere provision of a scheme of education, however complete in itself, is not sufficient, for many children are prevented by mental or physical incapacity from taking advantage of the education offered. The whole history of medical inspection of school children is a proof of this statement, and though England has been the latest of European, or indeed of most civilised countries to adopt it, medical inspection is the outcome of a general demand on the part of the nation (Sweden 1848 or earlier, Germany 1867, Denmark 1863, Austria 1873, Belgium 1874, France 1884, Japan 1894, Argentina 1894, Roumania 1899).

Following the Education Acts of 1870 and 1876 came the Blind and Deaf Children Act, 1893 (an obligatory Act, be it noticed), and the Defective Children (epileptic, feeble-minded, and cripples) Act of 1899. These legislative enactments have of necessity connoted the association of the physician with public education, an association which was bound to become more and more close by reason of the increasing recognition of the fact that these handicapped children are future citizens, and as such have the right to demand all the privileges which are associated with that state. (I include in this statement the feeble-minded, because it is clear from the provisions of this Act that the distinction between the feebly gifted children and the true feeble-minded was not clearly determined.) From this point of view it is but a small step to the provision of means for the amelioration of other conditions which exercise an adverse influence upon the physical fitness of the children of the nation, which, moreover, if unrelieved may produce a lifelong loss of efficiency.

What, then, are the defects discovered by medical inspection in the school? They may be roughly divided into three main groups.

(1) Sense—chiefly those of the eye and ear. (2) Contagious diseases which may be regarded as accidents rather than consequents of school attendance—ringworm, impetigo, ophthalmia, etc. (3) By far the most important group of defects are those due to conditions antecedent to or operating apart from school attendance—malnutrition, rickets, adenoids, etc.

To a large extent these defects are in a direct continuity of association with those conditions which lead to a high infant mortality rate and to a high death-rate of children under school age. It is, therefore, the extent of the facilities which already exist in Birmingham, and the degree to which they are utilised for the alteration of these defects, to which I wish to devote the few minutes at my disposal. The city is exceptionally well provided with voluntary hospitals.

Some idea of the numbers who require treatment may be gained from the following figures taken from my report for last year. 28,000 children were inspected (routine) in the area which now constitutes Greater Birmingham. Amongst these were found 3,000 having vision less than $\frac{6}{12}$, while there were 150 cases of corneal ulceration; 500 had other ocular defects; 2,300 children showed enlargement of tonsils and adenoids—570 children (in the former city only) were periodically inspected on account of ringworm, and I believe that at any given time there are at a moderate estimate 1,400 cases of ringworm in the enlarged city, and 400 children suffered from other skin complaints.

If we consider that there are 150,000 children in the elementary schools of the city, we can gain some idea of the numbers of school children who are at any time suffering from these and all the other ailments which bring children to the out-patient departments of hospitals. In this connection it must also be remembered that these out-patient departments draw their clientèle from a very wide area, extending more than twenty miles in all directions. A large amount of this increase is due directly to medical inspection in the counties. Given the number of remediable defects which are found, it will be conceded that it is to the interest of the community, the family, and the individual that such a remedy shall be found. It is difficult to form a reliable estimate of the number of children who follow up medical inspection with treatment, but inquiries in representative schools show that one-third of the total number found to have defects had received some form of treatment six months later. I am perfectly satisfied that parental indifference is but rarely the cause of the neglect to provide treatment in the remaining two-thirds. The difficulty of obtaining treatment through the private practitioner is obvious, for mostly the defects are those of the throat, ear, or eye, while any scheme of treatment on a provident basis is not likely to be successful so long as the hospitals continue to offer treatment free of charge to the individual requiring it, even though their resources are inadequate to meet the full demand for treatment.

But there are other disadvantages of leaning upon the voluntary hospitals of a practical kind.

#### Where the Hospitals are Wanting.

1. The hospitals are not organised for the treatment of many of the conditions which most require treatment amongst school children—*e.g.* discharging ears, ophthalmia, ringworm, etc., which require the daily ministrations of a nurse rather than of the doctor.

2. There is a great loss of time for the parents in waiting their turn for consultation.

3. The note system at present in vogue is unsatisfactory, as the facilities for obtaining notes vary greatly, and do not coincide with the urgency of the need for treatment. The system is, from another point of view, very unsatisfactory, in that it compels a form of begging which bears particularly hard upon parents who are not associated with some contributing fund.

4. Attendance at the hospital is often spasmodic and dependent upon the willingness of the parent to take the child there with regularity. The result is that there is no adequate supervision between the visits. Another difficulty is that some members of hospital staffs do not consider it part of their duty to state whether a child is or is not fit to go to school, while others, owing to the pressure of cases, or ignorance of the precise conditions which obtain in elementary schools, are tempted to sign a certificate that a child is unable to attend school without real or adequate reason. Now one of the main duties of a local Education Committee is to see that every child of school age is in attendance at school unless there is a reasonable cause for absence; in fact, it is the aim of a school medical officer to know the cause of absence on medical grounds in every case in which the absence is prolonged over three weeks.

The school medical officer is the centre of a number of agencies, which, though educational in character, are primarily intended to deal with the physical condition of the child—*e.g.* cripple schools, mentally defective, and epileptic centres, open-air schools, blind and deaf centres, etc. It is obvious that if the full value of these agencies is to be attained, it must be through a complete co-operation with, and the entire confidence of, the general body of medical practitioners engaged in private practice or in connection with charitable institutions. I believe I am right in stating that this co-operation is rapidly and steadily growing, for I now see quite a considerable number of children sent to me from hospitals and from private practitioners as suitable candidates for the open-air school and deaf centres, and the partially blind day class. Moreover, I am now in frequent communication with the almoner of the General Hospital in reference to cases which have come under her supervision. Another result of this mutual co-operation is the arrangement between the staffs of the voluntary hospitals (in large measure due to the energy of Mr. Howard Collins), whereby medical certificates for absence from school are signed and initialed by the member of the staff who has seen the case; so that should any question arise concerning attendance at school I am able to enter into communication with him or to re-examine the child independently.

There is one class of handicapped children for whom surgical treatment and special education must go hand in hand if satisfactory results are to be achieved. Speaking of England generally, treatment has been carried on, until recently, entirely independently of education, while education, on the other hand, has been entirely divorced from all attempts to assist the surgeon in his orthopædic efforts. It has been my good fortune to visit or to inquire into the organisation of cripple institutions in several countries in Europe and in America. Nothing is more striking than the complete collaboration which is to be found. This collaboration may even include other ameliorative agencies. Thus, in Copenhagen and several German institutes for cripples, bandages, surgical boots, irons, etc., are made by the cripples for the various clinics in the town.

It is idle to spend several years in effecting a cure of an orthopædic case if education is not given at the same time, while it is equally futile to attempt to educate a cripple if the opportunity of effecting a cure is allowed to slip by. There are signs that a beginning of this collaboration is being made in Birmingham.

Another sign of the increasing recognition of the valuable part which can be played by the school medical department is the attitude of other organisations dealing with the welfare of children. Thus the Charity Organisation Society, the City Aid Society, and the Women's Settlement frequently refer children to the school medical department, while a considerable number are examined for the Children's Country Holiday Fund. Numbers of children are also examined who are living in contact with notified cases of phthisis, while the names of all children notified as suffering from tuberculosis are reported from the Health Department for examination by the school medical officer. In this way a tuberculosis register has been founded, which contains some 400 names of children, many of whom are examined three and four times each year.

Mention should also be made of the co-operation of the school medical department with the work of the Advisory Committees of the Juvenile Labour Exchange. Should the school medical officer be of opinion that there are physical conditions in children about to leave school which contra-indicate any particular form of employment, this information is transmitted to the Advisory Committee. A similar interchange is being attempted with the certifying factory surgeon. Week by week I examine the intellectual capacities of children committed to the Remand Home.

Enough has been said to show that there is ample scope for useful work for the school medical department. What is still required is to place this relationship on a more definite footing, and to link up all these activities in such a way that the gaps into which so many children needing some form of help now fall may be filled up. I look forward to the time when the school medical department shall be a kind of central bureau of child welfare. In this connection we can learn a useful lesson from the organised work of the German schools.

The extraordinarily complete system of convalescent homes of all kinds, holiday camps, sanatoria, rest camps, advice in the choice of a trade, etc., which are in direct touch with the work of the school medical officer, show a unity of purpose which is lacking from our present hap-hazard methods. The Education Committee in its part has not been behindhand. Apart from special schools, an ophthalmic surgeon gives two mornings a week, and four dentists have now been appointed to attempt to deal with the problem of dental caries.

The Rev. E. D. Braimbridge (Kidderminster Infirmary):—

The point to which I wish to direct attention is a simple and practical one: How are we going to get, as working infirmary committees, sufficient money to carry on our institutions in future? In Kidderminster we find this increasingly difficult. One of our surgeons has urgently recommended that we should have a paid secretary whose work it should be to visit all likely subscribers, not only in the town but throughout the whole of the district. I should be very glad to know the practical experience of a paid secretary of any here. I should like to know Sir William Collins' opinion as to whether he would advocate putting voluntary hospitals on the rates.

Mr. G. H. Hume, Royal Victoria Infirmary, Newcastle-on-Tyne:—

I presume one reason why we are here is that each institution through its representative should, as it were, put its own individual experience at the service of the Association. I have been tempted thus early to intervene in the debate by the questions of the last speaker. In Newcastle we have several times tried the expedient of a paid secretary, and have always given it up because we found that it failed. People who will not voluntarily give will not readily give when visited or importuned for money. At the present moment we are largely dependent, and we are becoming more and more dependent as time goes on, upon the subscriptions of the working men in the district. The expenditure of the Royal Victoria Infirmary is, speaking roughly, £36,000 a year, and of that amount £20,000 is supplied by the working men. We have to encroach upon the legacies fund to a very considerable extent, and we think it sufficiently good business if upon the annual balance-sheet there is a considerable sum which remains to be invested. On that principle, well recognised in England, that where money is contributed, power is expected, the working men claim a very large representation upon the governing body. That is attended by certain advantages, and also certain disadvantages. In the first place it has unduly enlarged the governing body, which is the House Committee.

The Chairman intervened to remind the speaker that the discussion must be confined to the paper before the Conference.

Mr. Hume (continuing) : The subject of the paper was "Hospitals and the State." One reason why hospitals will come under State control is for want of financial support. So long, I believe, as the hospitals obtain support each from its own community there will be very little chance of the State intervening and taking over the management. I think, therefore, that the only way to avoid State intervention is to have each community sufficiently interested in the support of its own hospital. I am old enough to have had experience of a governing body or House Committee of the old style, consisting of a small number of the practical business and leisured men of the town, and I have also had experience of the democratic government of a hospital, and I know that now there is much less difficulty in getting alterations and improvements made.

Mr. J. H. Cooke, Albert Infirmary, Winsford, Cheshire:—

It is very difficult for anyone to decide the question of whether it is advisable for the hospitals to be taken over by the State, because in this country, so far as I know, no practical experience of that character has been obtained. Immediately you have got State intervention in the matter of the hospitals you have done away with the sympathy, to a large extent, of the generous public, and you create strife and difficulties. The interference of the State will to a large extent do away with a valuable part of the work carried on in country hospitals, especially where ladies' committees go round and sympathise with the patients. Now, may I introduce a personal remark with regard to education? Thirty years ago, in Cheshire, we started evening classes with twenty-three students, and worked them up to 600, all the work being honorary, except in the case of the teachers. Then the Education Act came into force. Of course we wanted money, but I am bound to say that since the Education Act came into operation and we have received more money, the schools, instead of going up, have gone down. This shows distinctly that the more State interference you have the less local sympathy is aroused.

Colonel Roxburgh, Chairman of the House Committee, Western Infirmary, Glasgow:—

The vast majority of those present are in favour of the voluntary hospital, and we all believe that if we were let alone we should be able to continue the voluntary principle. The paper to which we have listened is a very interesting one to those endeavouring to manage voluntary hospitals, especially in large centres of population, where the question of the number of children attending the out-patient departments is becoming a very pressing one. How do those present propose to deal with it? In our out-patient department at Glasgow the number of throat and nose cases has increased and is putting a great amount of extra work upon the staff there. Our School Board has got a share of the money spoken of in the paper, but a very small sum—£700—and I understand that for the time being they are devoting that entirely to dental treatment, so that there is nothing whatever for the other diseases which are found out by the doctors who inspect the children. One of our institutions has taken a lump sum, but it has been found to be very unsatisfactory, and does not anything like meet the extra cost to which they are put. We in our hospital have declined to take any lump sum, and in the meantime we are feeling our way as to what the exact amount of extra work put upon us is to be. Our idea is that if we could get the School Board to establish their own clinic we might quite easily give them the services of certain members of our staff, who, of course, would be paid by them, and give them the use of such accommodation as they might require, as it was not wanted by ordinary patients. It would be interesting to hear the experience of hospital managers in other parts of the country in connection with this very important matter.

Mr. C. A. Mason, Birmingham Eye Hospital:—

Dr. Auden, I take it, got up rather to answer this paper, and he did not make any attempt to justify the criticism that the education authorities have not endeavoured to co-operate with the voluntary hospitals, but have rather aimed at establishing their own clinics, which really seems to be a more expensive method. In addition, the paper draws our attention to the almost inevitable trend that hospitals will not be able to be carried on much longer in their present voluntary state. It seems to me that Sir William Collins suggests that if a sympathetic attitude were adopted by the State authorities towards the voluntary hospitals that they would be able to maintain their own present individuality and yet be able to serve the State, perhaps, more efficiently than by the various units of the State setting up their particular clinics.

[This debate was followed by Mr. Edgar S. Kemp's paper on "The Training and Work of the Almoner," which, with the discussion that ensued upon it and the rest of the proceedings, we shall publish next week.—Ed. The Hospital.]

